# Special Issue: Antimicrobial Resistance in Livestock

**DOI:** 10.3390/microorganisms8050645

**Published:** 2020-04-28

**Authors:** Kim Stanford

**Affiliations:** Agriculture Centre, Alberta Agriculture and Forestry, Lethbridge, AB T1J 4V6, Canada; Kim.Stanford@gov.ab.ca

Antimicrobial resistance threatens the health of both humans and livestock as antimicrobials become continually less effective for controlling infectious disease. However, much is unknown about the extent of the transfer of antimicrobial-resistant bacteria from livestock to humans or vice versa. More information is also needed on the relationship between antimicrobial resistance and food safety, the extent to which antimicrobial resistance is influenced by antimicrobial use, or even how antimicrobial resistance should be assessed for specific pathogens. Antimicrobial resistance is a global issue, but has a differential impact depending on national practices in human and veterinary health, food safety, and the establishment and enforcement of regulations that control the use of antimicrobials. This Special Issue contains nine articles dealing with these key issues and demonstrates the global impact of antimicrobial resistance in livestock, with articles submitted from Africa, Asia, North and South America. 

In order to compare antimicrobial resistance over time and across locations, standard methods for evaluating antimicrobial sensitivity are required. A recent study outlines a new method for determining the antimicrobial sensitivity of *Mycoplasma bovis*, a pathogen becoming increasingly prevalent in bovine respiratory disease [1]. Recent work with extended-spectrum β lactamase-producing *E. coli*, has demonstrated that these high-priority pathogens have distinct lineages when comparing human and cattle strains [2]. In contrast, antimicrobial-resistant *Brucella* spp. are likely being transferred from livestock to humans and vice versa, making it challenging to treat brucellosis in humans, an endemic disease in Egypt [3]. Although antimicrobial resistance in bovine respiratory disease organisms in cattle was reduced in those managed without the use of antimicrobials, the origin of cattle also influences antimicrobial resistance. A *Mannheimia haemolytica* strain resistant to the majority of antimicrobials was first isolated at only one feedlot, but then rapidly spread to multiple locations [4]. The use of antimicrobials as growth promotors in livestock has been curtailed by legislative changes in some jurisdictions and the use of chlortetracycline as a growth promoter increased tetracycline resistance in *Campylobacter jejeuni*, although Canadian beef products were not contaminated by resistant bacteria and food safety risks were minimal [5]. In Brazil, *Listeria monocytogenes* were present in 12% of beef products sampled and, while antimicrobial resistance was rare, strains were resistant to a common sanitizer used to clean processing equipment, posing a risk to meat hygiene and human health [6]. In China, carbapenem and colistin-resistant *E. coli* were isolated from pig farms [7]. This resistance was plasmid-based, and during conjugation assays the transfer of multiple antimicrobial resistance genes generated multi-drug resistant *E. coli*. On further study of this population of Chinese pigs, an *E. coli* with chromosomally encoded *mcr-1*, along with a suite of antimicrobial resistance genes on plasmids, was isolated [8]. When evaluating *E. coli* isolated from South African cattle, the antimicrobial resistance detected was largely to tetracycline and was not considered a human health risk [9]. However, some antimicrobial-resistant strains of South African *E. coli* also formed extremely strong biofilms, markedly increasing their risk to food safety. 

Many thanks to the members of the *Microorganisms* editorial office for their help in managing and organizing this Special Issue, and for giving me this opportunity. Thank you also to the authors and reviewers for their excellent work.
